# Differences in the molecular signatures of mucosal-associated invariant T cells and conventional T cells

**DOI:** 10.1038/s41598-019-43578-9

**Published:** 2019-05-08

**Authors:** Daeui Park, Hong Gi Kim, Miok Kim, Tamina Park, Hyung-Ho Ha, Dae Ho Lee, Kang-Seo Park, Seong Jun Park, Hwan Jung Lim, Chang Hoon Lee

**Affiliations:** 1 Department of Predictive Toxicology, Korea Institute of Toxicology, Daejeon, Korea; 2 Center for Convergent Research of Emerging Virus Infection, Korea Research Institute of Chemical Technology (KRICT), Daejeon, Korea; 3 Center for Information-Based Drug Research, Bio and Drug Discovery Division, Korea Research Institute of Chemical Technology, Daejeon, 34114 Republic of Korea; 4 College of Pharmacy, Suncheon National University, Suncheon, Republic of Korea; 5 Department of Oncology, Asan Medical Center, University of Ulsan College of Medicine, Seoul, 05505 Republic of Korea

**Keywords:** Immunogenetics, Gene regulatory networks, Immunogenetics

## Abstract

Mucosal-associated invariant T (MAIT) cells exhibit different characteristics from those of TCRα7.2^−^ conventional T cells. They play important roles in various inflammatory diseases, including rheumatoid arthritis and inflammatory bowel disease. MAIT cells express a single T cell receptor alpha chain, TCRα7.2 segment associated with Jα33 and CDR3 with fixed length, which recognizes bacteria-derived vitamin B metabolites. However, the characteristics of MAIT cells and TCRα7.2^+^ CD161^−^ T cells have never been compared. Here, we performed RNA sequencing to compare the properties of MAIT cells, TCRα7.2^−^ conventional T cells and TCRα7.2^+^ CD161^−^ T cells. Genome-wide transcriptomes of MAIT cells, TCRα7.2^−^ conventional T cells, and TCRα7.2^+^ CD161^−^ T cells were compared and analyzed using causal network analysis. This is the first report comparing the transcriptomes of MAIT cells, TCRα7.2^−^ conventional T cells and TCRα7.2^+^ CD161^−^ T cells. We also identified the predominant signaling pathways of MAIT cells, which differed from those of TCRα7.2^−^ conventional T cells and TCRα7.2^+^ CD161^−^ T cells, through a gene set enrichment test and upstream regulator analysis and identified the genes responsible for the characteristic MAIT cell phenotypes. Our study advances the complete understanding of MAIT biology.

## Introduction

T cells are critical to the diverse regulation of the immune system. They are also an essential component in the reaction to invading pathogens. Effector functions of T cells in diverse immunological processes are determined by the biological characteristics of T cells, such as their highly selective recognition of peptide antigens using very diverse T cell receptors (TCRs) restricted by MHC class I or MHC class II, and their differentiation into diverse lineages. However, recently identified T cell subsets, such as natural killer T cells (NKT cells) and mucosal-associated invariant T (MAIT) cells, have challenged common ideas about T cell biology^1^. NKT cells recognize foreign lipids and glycolipids restricted by nonpolymorphic CD1d molecules. MAIT cells recognize bacterial-derived vitamin B metabolites restricted by the MHC class I-like protein MR1^2–5^. These antigens are beyond the boundaries of those recognized by conventional T cells, which have highly diverse TCR molecules restricted by MHC class I or MHC class II. Thus, NKT cells and MAIT cells broaden the sensory range of pathogens or foreign antigens in addition to conventional T cells^6,7^. MAIT cells were identified most recently, and their immunological functions are not yet fully understood. In our previous study, we demonstrated that MAIT cells are a critical inflammatory component in rheumatoid arthritis^8^. Other recent studies have revealed the critical role of MAIT cells in various inflammatory diseases, including ulcerative colitis, psoriasis and several cancers. This is in addition to their role in defeating bacterial pathogens^9–12^. The characteristics of MAIT cells, which have effector functions distinguishable from those of conventional T cells, are unclear.

In our recent study^13^, we found that the C/EBP/δ-mediated transcriptional pathway in MAIT cells involves highly expressed inflammatory adhesion molecules, which include C-C chemokine receptor type 6 (*CCR6*), fucosyltransferase 7 (*FUT7*), and ST3 beta-galactoside alpha-2,3-sialyltransferase 4 (*ST3GAL4*). The molecules participate in the synthesis of the sialic Lewis acid motif-glycosylation modification of selectin ligands. This study was the first report of a transcription factor, C/EBP/δ, in T cells. Only MAIT cells express high levels of C/EBP/δ protein to at least partially acquire MAIT cell functions. We also confirmed the presence of highly upregulated C/EBP/δ in MAIT cells. Although there have been several important studies, such as our recent study^13^, that have explored the MAIT cell phenotype, information on the specific genes necessary for the MAIT phenotype has been lacking until now.

In the present study, we performed genome-scale analysis of gene expression profiles using RNA sequencing (RNA-Seq) based on next-generation sequencing methods. This was done to better understand the potential causative molecular characteristics of the unique cellular effector functions of MAIT cells, which differ from conventional T cells. We identified previously unreported genes (*SLCA4A10*, *SCART1*, *WNT11*, *LTK*, *FLT4*, *LEF1*, *KLRC4*, *TBC1D4*, *LAIR2*, *KLRB1* (encodes CD161), *ZFP36L2*, *DUSP2*, *ARL4C*, *SNORA70*, *LDHB*, *ITK*, *FYB*, *MIAT*, and *TMEM173*, among others) that may determine the characteristics of MAIT cells. We also characterized the predominant signaling pathways of MAIT cells through Ingenuity Pathway Analysis (IPA) and gene ontology (GO) analysis, which have not been described before. We attempted to characterize TCRα7.2^+^ CD161^−^ T cells in comparison to MAIT cells. Although the existence and non-MAIT cell-like characteristics of TCRα7.2^+^ CD161^−^ T cells have been recognized^8^, we did attempt to uncover the characteristics and relationships of TCRα7.2^+^ CD161^−^ T cells with MAIT cells. RNA-seq analysis was performed to compare the similarities and differences between MAIT cells and TCRα7.2^+^ CD161^−^ T cells. The results reveal a marked difference between TCRα7.2^+^ CD161^−^ T cells and MAIT cells, with the former being nearly identical to conventional T cells. The findings advance the knowledge of MAIT cells.

## Results

### Differences between the gene expression profiles of MAIT, TCRα7.2^+^ CD161^−^ T cells, and TCRα7.2^−^ conventional T cells

**Figure 1:**
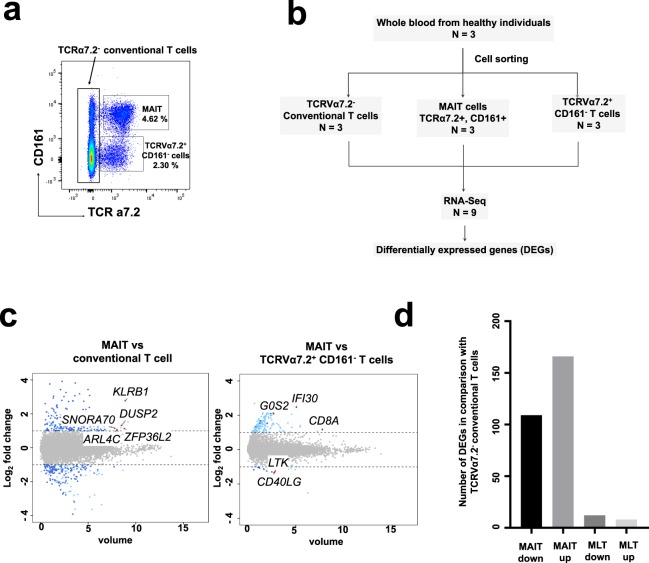
Gene expression profiles of MAIT cells, TCRα7.2^+^ CD161^−^ T cells, and TCRα7.2^+^ conventional T cells. (**a**) Frequencies of TCR V_α_7.2^+^ CD161^+^ MAIT cells, TCR V_α_7.2^+^ CD161^−^ T cells and conventional T cells isolated from peripheral blood (PB) of healthy donors. Representative dot plots from 10 healthy donors are shown. (**b**) The strategy to sort TCR V_α_7.2^+^ CD161^+^ MAIT cells, TCR V_α_7.2^+^ CD161^−^ T cells and conventional T cells isolated from peripheral blood from three different healthy donors for RNA-Seq analysis. (**c**) Scatter dot plot indicating differentially expressed genes (DEGs) between MAIT vs. TCRα7.2^+^ conventional T cells and MAIT vs., TCRα7.2^+^ CD161^−^ T cells. The Y axis shows fold changes in expression level (Log2 value), and the X axis depicts volume. The volume indicates the level of gene expression. The volume was calculated by geometric means of mapped reads between two conditions. (**d**) Number of upregulated and downregulated DEGs in MAIT and TCRα7.2^+^ CD161^−^ T cells in comparison with TCRα7.2^−^ conventional T cells. DEGs were selected by a fold change cut-off of >2 and p-value < 0.05.

To better understand the differentiating characteristics between MAIT cells and conventional T cells, we determined gene expression profiles on a genome-wide scale. We first stained CD3^+^ T cells with TCRa7.2 and CD161, which are markers of MAIT cells, to distinguish human MAIT cells from non-MAIT cells. This was achieved through the identification of MAIT cells as TCRa7.2 and CD161 double-positive cells (Fig. 1a). Conventional T cells (TCRa7.2− T cells) and TCRa7.2+ and CD161− T cells were present in non-MAIT T cells (Fig. 1a). We analyzed the similarities and differences among TCRα7.2^−^ conventional T cells, MAIT cells, and TCRα7.2^+^ CD161^−^ T cells using RNA-Seq based on next-generation sequencing. Conventional T cells (TCRa7.2^−^ CD3^+^ T cells), MAIT (TCRa7.2+ and CD161+ CD3+ T cells), and TCRα7.2^+^ CD161^−^ T cells from healthy human peripheral blood were separated using FACS (Fig. 1b). Comparison of the gene expression profiles of MAIT vs. conventional T cells and MAIT vs. TCRα7.2^+^ CD161^−^ T cells (Fig. 1c) revealed that MAIT cells differed markedly from conventional T cells and from TCRα7.2^+^ CD161^−^ T cells. RNA-seq analysis revealed 275 differentially expressed genes (DEGs) between MAIT and conventional T cells and only 20 DEGs between TCRα7.2^+^ CD161^−^ T cells and conventional T cells. Of the 275 DEGs between conventional and MAIT cells, 166 genes were significantly upregulated, and 109 were significantly downregulated. Compared to conventional T cells, in TCRα7.2^+^ CD161^−^ T cells, 12 genes were significantly downregulated, and eight genes were significantly upregulated (Fig. 1d). To elucidate the differences between MAIT cells and conventional T cells, we chose the top five upregulated and top five downregulated genes in MAIT cells compared to TCRα7.2^−^ conventional T cells (Table 1). The *SLCA4A10*, *SCART1*, *WNT11*, *LTK*, and *FLT4* genes were upregulated in MAIT cells 15.10, 14.10, 13.57, 10.86, and 10.78 times, respectively, compared to TCRα7.2^−^ conventional T cells. These genes were highly enriched in volume, indicating that they might play an important role in the characterization of MAIT cells. *LEF1*, *CCR7*, *KLRC4*, *TBC1D4*, and *LAIR2* genes were downregulated −15.01, −9.15, −6.87, −6.66, and −6.27 times, respectively, in MAIT cells compared to TCRα7.2^−^ conventional T cells. These genes were also highly enriched in volume, indicating a large amount of expression. The top 10 genes with the greatest differences in TCRα7.2^+^ CD161^−^ T cells and TCRα7.2^−^ conventional T cells were completely different from those of MAIT and TCRα7.2^−^ conventional T cells, strongly suggesting that TCRα7.2^+^ CD161^−^ T cells are different from MAIT (Table 1). We also analyzed five upregulated DEGs and five downregulated DEGs with the highest volume values among DEGs between MAIT and TCRα7.2^−^ conventional T cells. The volume values of the *KLRB1* (encoding CD161), *ZFP36L2*, *DUSP2*, *ARL4C*, and *SNORA70* genes were the highest (8.90, 8.79, 8.50, 8.01 and 7.79, respectively). *LDHB*, *ITK*, *FYB*, *MIAT*, and *TMEM173* showed volume values of 7.73, 6.00, 5.63, and 4.92, respectively. In particular, the *KLRB1* gene was highly differentially expressed because MAIT cells were sorted by the CD161 marker. These genes were upregulated or downregulated by a factor greater than 2. The five upregulated and five downregulated DEGs displaying the highest volume among DEGs between TCRα7.2^+^ CD161^−^ T cells and TCRα7.2^−^ conventional T cells also differed from those of MAIT cells, strongly suggesting that TCRα7.2^+^ CD161^−^ T cells are different from MAIT cells (Table 2).

**Table 1:** Highly differentially expressed genes sorted by fold change.

Gene Symbol	Gene Description	Fold Change	Volume
**MAIT vs. TCRα7.2 ^**−**^ **conventional T cells**			
*SLC4A10*	solute carrier family 4, sodium bicarbonate transporter, member 10	15.10	2.27
*SCART1*	scavenger receptor protein family member	14.10	3.64
*WNT11*	wingless-type MMTV integration site family, member 11	13.57	0.97
*LTK*	leukocyte receptor tyrosine kinase	10.86	4.98
*FLT4*	fms-related tyrosine kinase 4	10.78	2.10
*LEF1*	lymphoid enhancer-binding factor 1	−15.01	3.40
*CCR7*	C-C chemokine receptor type 7 isoform c precursor	−9.14	3.55
*KLRC4*	killer cell lectin-like receptor subfamily C, member 4	−6.87	2.66
*TBC1D4*	TBC1 domain family, member 4	−6.66	2.34
*LAIR2*	leukocyte-associated immunoglobulin-like receptor 2	−6.27	2.42
**TCRα7.2 ^**+**^ **CD161** ^**−**^ **T cells vs. TCRα7.2** ^**−**^ **conventional T cells**			
*IFI30*	interferon, gamma-inducible protein 30	5.55	5.06
*TNFAIP2*	tumor necrosis factor, alpha-induced protein 2	4.28	2.38
*G0S2*	G0/G1 switch 2	4.25	2.69
*RAB32*	RAB32, member RAS oncogene family	3.20	1.33
*MAFB*	v-maf avian musculoaponeurotic fibrosarcoma oncogene homolog B	2.89	1.26
*TNFRSF4*	tumor necrosis factor receptor superfamily, member 4	−3.41	2.66
*CCR6*	chemokine (C-C motif) receptor 6	−3.12	1.94
*CD40LG*	CD40 ligand	−2.52	2.74
*TNFRSF18*	tumor necrosis factor receptor superfamily, member 18	−2.45	2.50
*LTK*	leukocyte receptor tyrosine kinase	−2.42	2.84

**Table 2:** Highly differentially expressed genes sorted by volume.

Gene Symbol	Gene Description	Fold Change	Volume
**MAIT vs. TCRα7.2 ^**−**^ **conventional T cells**			
*KLRB1(CD161)*	killer cell lectin-like receptor subfamily B, member 1	6.82	8.90
*ZFP36L2*	ZFP36 ring finger protein-like 2	2.20	8.79
*DUSP2*	dual specificity phosphatase 2	2.49	8.50
*ARL4C*	ADP-ribosylation factor-like 4C	2.08	8.01
*SNORA70*	small nucleolar RNA, H/ACA box 70	2.23	7.79
*LDHB*	lactate dehydrogenase B	−2.15	7.73
*ITK*	IL2-inducible T cell kinase	−2.44	6.00
*FYB*	FYN binding protein	−2.8	5.63
*MIAT*	myocardial infarction associated transcript (nonprotein coding)	−2.12	5.27
*TMEM173*	stimulator of interferon genes protein isoform 2	−2.02	4.91
**TCRα7.2 ^**+**^ **CD161** ^**−**^ **T cells vs. TCRα7.2** ^**−**^ **conventional T cells**			
*IFI30*	interferon, gamma-inducible protein 30	5.55	2.38
*G0S2*	G0/G1 switch 2	4.25	2.69
*TNFAIP2*	tumor necrosis factor, alpha-induced protein 2	4.28	1.33
*TMEM176B*	transmembrane protein 176B	2.87	1.26
*LTK*	leukocyte receptor tyrosine kinase	−2.40	2.66
*CD40LG*	CD40 ligand	−2.52	1.94
*WNT7A*	wingless-type MMTV integration site family, member 7A	−2.02	2.74
*TNFRSF4*	tumor necrosis factor receptor superfamily, member 4	−3.41	2.50
*TNFRSF18*	tumor necrosis factor receptor superfamily, member 18	−2.45	2.84

### Gene set analysis among MAIT cells, TCRα7.2^+^ CD161^−^ T cells, and TCRα7.2^−^ conventional T cells and transcription factor analysis

**Figure 2:**
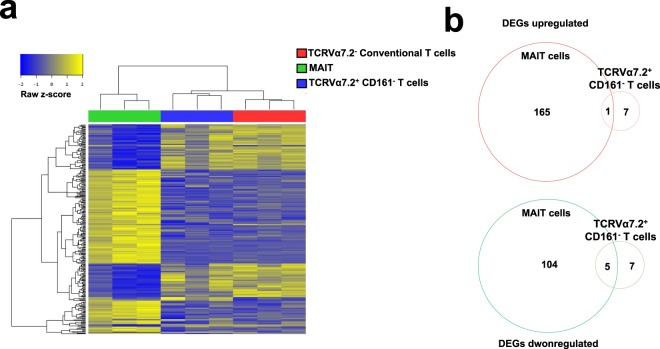
TCR V_α_7.2^+^ CD161^−^ T cells have different gene expression patterns than MAIT cells. (**a**) Heat map of the two-way hierarchical clustering of MAIT cells, TCRα7.2^+^ CD161^−^ T cells and TCRα7.2^+^ conventional T cells isolated from peripheral blood (PB) of three different healthy donors (285 genes with a greater than fold change and Z-score for normalized value were used; log2-based). All experiments of each condition were replicated three times. (**b**) Venn diagram of upregulated and downregulated differentially expressed genes (DEGs) between MAIT cells and TCRα7.2^+^ CD161^−^ T cells compared to TCRα7.2^−^ conventional T cells. A detailed list of the genes in the Venn diagram is provided in Supplementary Fig. S1.

**Figure 3:**
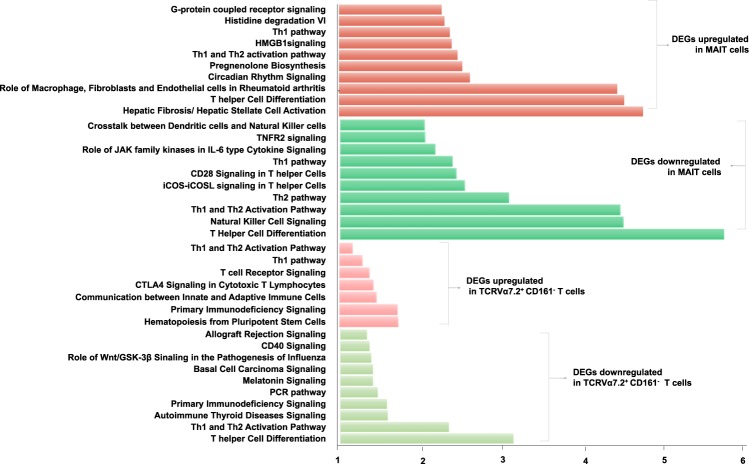
Gene set enrichment test. (**a**) Gene set analysis based on the DEGs derived from RNA-Seq analysis is shown. Top 10 gene sets of DEGs upregulated in MAIT cells, top 10 gene sets of DEGs downregulated in MAIT cells, total 7 gene sets of DEGs upregulated in TCRα7.2^+^ CD161^−^ T cells and 10 gene sets of DEGs downregulated in TCRα7.2^+^ CD161^−^ T cells.

In our RNA-Seq analysis, the genomic expression of MAIT cells and CD161^−^ conventional T cells was very different. Although TCRα7.2^+^ CD161^−^ T cells commonly express TCRα7.2, the most important marker of MAIT cells, they exhibit almost the same gene expression pattern as CD161^−^ conventional T cells and show a very large difference from MAIT cells. The differential gene expression patterns of MAIT cells and TCRα7.2^+^ CD161^−^ T cells were confirmed via a heat map in this study. The heat map for all genes (285 genes) with a fold change of more than +/−2 in MAIT and TCRα7.2^+^ CD161^−^ T cells, compared to TCRα7.2^−^ conventional T cells, is shown in Fig. 2a. As shown in the heat map, MAIT cells are clearly different from TCRα7.2^−^ conventional T cells in their gene expression profiles, but TCRα7.2^+^ CD161^−^ T cells are almost similar to those of CD161^−^ conventional T cells (Fig. 2a). A Venn diagram was constructed using only upregulated or downregulated genes, identifying only one gene that was upregulated in both MAIT cells and TCRα7.2^+^ CD161^−^ T cells when compared to CD161^−^ conventional T cells (Fig. 2b). We present a list of 165 genes that were upregulated only in MAIT cells, as well as a list of 7 genes that were upregulated only in TCRα7.2^+^ CD161^−^ T cells (Supplemental Fig. S1). Only one gene, Adrenomedullin (*ADM*), was upregulated in both MAIT and TCRα7.2^+^ CD161^−^ T cells (Supplemental Fig. S1). Compared to CD161^−^ conventional T cells, in MAIT and TCRα7.2^+^ CD161^−^ T cells, 5 genes were commonly downregulated (Fig. 2b). The 5 genes were *TNFRSF4*, *FBLN7*, *KIR2DS4*, *WNT7A*, and *ANKRD55* (Supplemental Fig. S1). We present a list of 104 genes that were downregulated only in MAIT cells, as well as a list of 7 genes that were downregulated only in TCRα7.2^+^ CD161^−^ T cells (Supplemental Fig. S1). Based on the DEGs derived from RNA-Seq analysis, we performed gene set enrichment analysis to infer the functional differences between MAIT and TCRα7.2^+^ CD161^−^ T cells compared to TCRα7.2^−^ conventional T cells. We analyzed the 10 gene sets with the most significant P-values via the upregulated and downregulated DEGs in the MAIT and TCRα7.2^+^ CD161^−^ T cells compared to TCRα7.2^−^ conventional T cells (Fig. 3). The top 10 gene sets of MAIT cells compared to TCRα7.2^−^ conventional T cells were clearly different from those of TCRα7.2^+^ CD161^−^ T cells. For the upregulated genes, MAIT and TCRα7.2^+^ CD161^−^ T cells were enriched with different categories, except for the Th1 and Th2 signaling pathways. We can also see that all the downregulated genes, except for Th1 and Th2 signaling pathways, are enriched with different sets. These results show that MAIT cells are distinct from TCRα7.2^−^ conventional T cells and are different from TCRα7.2^+^ CD161^−^ T cells. The transcription factors could be regarded as the distinguished regulators between MAIT cells and TCRα7.2^−^ conventional T cells. In upregulated genes, the factors are cAMP Responsive Element Modulator (*CREM*), Inhibitor of DNA Binding 2 (*ID2*), Zinc Finger And BTB Domain Containing 16 (*ZBTB16*), and CCAAT Enhancer-Binding Protein Delta (*CEBPD*), which showed fold changes of 2.84, 2.37, 8.43, and 9.11, respectively. In downregulated genes, the factors are Forkhead Box P3 Scurfin (*FOXP3*) and Inhibitor of DNA Binding 3 (*ID3*), which showed fold changes of −3.65 and −2.54, respectively. ID2/3 proteins have been shown to promote the differentiation of conventional αβ and γδT cells and to suppress the expansion of invariant natural killer T (iNKT) cells and innate-like γδNKT 21 within their respective cell lineages. Additionally, ID3-deficient (ID3−/−) mice support the expansion of innate-like γδNKT cells^14^. Interestingly, ID2 and ID3 were reversely expressed in MAIT cells. Although we could not conclude that the differentiation of MAIT cells needs both the upregulation of ID2 and downregulation of ID3, this result suggests that ID2 and ID3 potentially have some roles in MAIT cells, which need to be further studied.

### Differential signaling pathways between MAIT and conventional T cells

**Figure 4:**
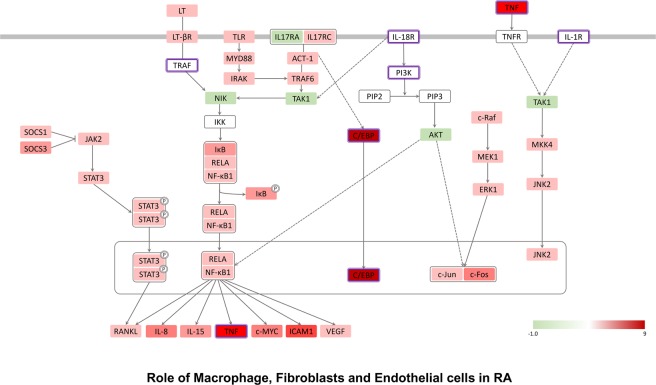
Role of macrophage, fibroblast and endothelial cell pathways in MAIT cells. IPA analysis of DEGs of MAIT cells in comparison with conventional T cells showed a functionally activated MAIT cell signaling pathway in association with macrophage, fibroblast and endothelial cell roles in rheumatoid arthritis. MAIT cells regulate NF-kB activity through TLRs and LT-BRs in the innate immune system while also regulating the NF-kB signaling pathway through receptors for IL-17 cytokines. The box indicates the gene involved in the biological pathway. The red box represents upregulated genes, but the green box shows downregulated genes. The purple box indicates already known important genes in each biological pathway. The information was obtained from the IPA database. A black (white) box indicates that there is no expression data from the RNA-seq experiments. Solid lines provide evidence of the directed interaction between two genes. However, the dotted line indicates the in-directed interaction.

**Figure 5:**
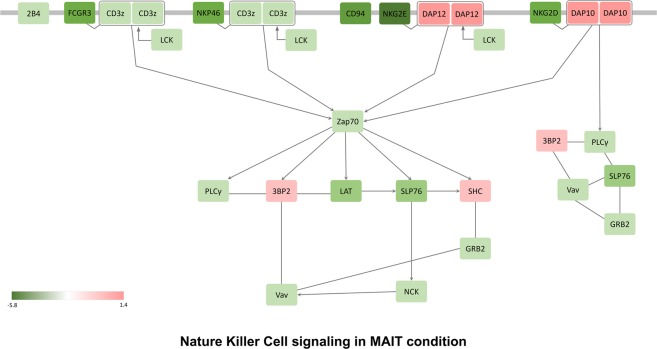
Natural killer cell signaling pathway of MAIT cells in comparison with conventional T cells. IPA analysis of DEGs of MAIT cells in comparison with conventional T cells showed a functionally activated signal pathway of MAIT cells in association with the role of natural killer cell signaling. In MAIT cells, almost all NK cell-specific genes were downregulated. For example, CD94, NKG2D, and NKG2D are all downregulated significantly, indicating that MAIT cells are very different from NK cells.

**Figure 6:**
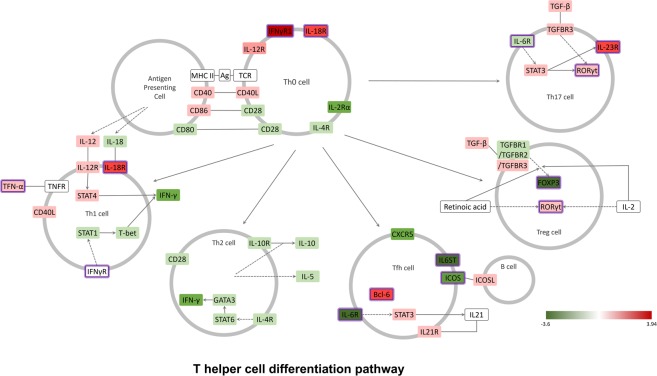
T helper cell differentiation pathway of MAIT cells in comparison with conventional T cells. IPA analysis of DEGs of MAIT cells in comparison with conventional T cells showed a functionally activated MAIT cell signaling pathway in association with the role of the T helper cell differentiation pathway. The T helper cell differentiation pathway changed significantly in MAIT cells as the Th17 phenotype increased. Additionally, the RORγt transcription factor was greatly increased, and the IL23 receptor was also increased. The strong expression of Th17 lineage-related RORγt and related genes, such as IL-23R, was significantly upregulated in MAIT cells. These results strongly suggest that MAIT cells possess innate cell characteristics and Th17-associated adaptive T cell characteristics.

IPA analysis revealed similar signaling routes of macrophages, fibroblasts, and endothelial cell pathways related to innate immunity in MAIT cells (Fig. 4). The IL-17 receptors *IL-17Ra* and *IL-17Rc* were significantly altered, as were the *ACT-1*, tumor necrosis factor receptor-associated factor 6 (*TRAF6*) and transforming growth factor beta-activated kinase 1 (*TAK1*) signaling pathways. Lympho-toxin beta receptors (*LT-BR*) and Toll-like receptors (*TLR*), which are receptors for LT and various innate immune activating TLR ligands, were also important in MAIT cells (Fig. 4). Myeloid differentiation primary response 88 (*MYD88*), interleukin-1 receptor-associated kinase (*IRAK*), nuclear factor-kappa-B-inducing kinase (*NIK*) and nuclear factor-kappa B (*NF-kB*) signal pathway components were significantly increased. *IL-15*, *RANKL*, and vascular endothelial growth factor expression levels were also increased in MAIT cells compared to conventional T cells. Overall, MAIT cells might regulate NF-kB activity through TLRs and LT-BRs in the innate immune system while also regulating the NF-kB signaling pathway through receptors for IL-17 cytokines (Fig. 4). These transcriptome results were also supported by previous reports^15,16^. Salio *et al*.^16^ showed that TLR agonists such as LPS and R848 induced MAIT cell signaling. Additionally, the NF-kB signaling pathway was demonstrated to be a critical signaling component in MAIT cell differentiation^15^. We also showed that the IL-17 receptor-C/EBP signal pathway was highly increased in MAIT cells. This result might be consistent with our previous report of an important role of C/EBP protein in MAIT cell function^13^. NK cell signaling pathways were downregulated in MAIT cells. In MAIT cells, almost all NK cell-specific genes were downregulated (Fig. 5). For example, *CD94*, *NKG2D*, and *NKG2D* are all significantly downregulated, indicating that MAIT cells are very different from NK cells (Fig. 5). We also confirmed that the T helper cell differentiation pathway changed significantly in MAIT cells as the Th17 phenotype increased (Fig. 6). The RORγt transcription factor was greatly increased, and the IL23 receptor was also increased (Fig. 6).

## Discussion

MAIT cells express TCRs and have innate-like characteristics in comparison with conventional T cells. The importance of MAIT cells has become increasingly relevant. Recent studies, including our own, have indicated that MAIT cells could play a vital role in a variety of inflammatory diseases^8–12^. The genetic and biological characteristics of MAIT cells remain unclear. In this study, RNA-Seq analysis of the entire genome was performed to conclusively differentiate MAIT cells from CD161^−^ conventional T cells and TCRα7.2^+^ CD161^−^ T cells, although the level of gene expression for each gene should be carefully evaluated by various experiments.

The gene expression profile of TCRα7.2^+^ CD161^−^ T cells was similar to that of conventional T cells. Compared to conventional T cells, in MAIT cells, a number of genes were significantly changed, as expected. The expression of very few genes was altered in TCRα7.2^+^ CD161^−^ T cells. In addition, the majority of the genes that differed between TCRα7.2^+^ CD161^−^ T cells and conventional T cells were not genes that differed in MAIT cells from conventional T cells. This finding suggests that TCRα7.2^+^ CD161^−^ T cells are markedly different from MAIT cells. TCRα7.2^+^ CD161^−^ T cells differ from MAIT cells in terms of their similarity in the differentiation process, even though they express TCRα7.2^8,13^. Further research is needed to determine whether there is a systematic commonality and to define the fundamental basis of TCRα7.2^+^ CD161^−^ T cells and their role(s). Whether TCRα7.2^+^ CD161^−^ T cells are undifferentiated MAIT cells is unknown. Presently, GO analysis of DEGs demonstrated strong innate immunity-associated characteristics of MAIT cells (Fig. 3). Signaling pathways involving macrophages and fibroblasts display many similarities. These two cell types are important innate cell components in various immune-related diseases and antimicrobial defense systems (Fig. 4) and are a component of antitumoral and antiviral immunity^17–19^. These findings suggest that MAIT cells and NK cells are effector components equipped with differential signaling or sensing systems to broaden a range of host defenses. Interestingly, MAIT cells are mainly considered a key player in antibacterial defense, rather than antivirus or tumor defense^20^, although several recent studies also show potential activities in antiviral immunity^21,22^.

We also confirmed that the strong expression of Th17 lineage-related RORγt and related genes, such as IL-23R, is significantly upregulated in MAIT cells (Fig. 5)^23,24^. These results strongly suggest that MAIT cells possess innate cell characteristics and Th17-associated adaptive T cell characteristics.

The study includes novel findings that the MAIT cell genes *SLCA4A10*, *SCART1*, *LTK*, *FLT4*, *LEF1*, *KLRC4*, *TBC1D4*, *LAIR2*, *ZFP36L2*, *KLRB1*, *DUSP2*, *ARL4C*, *SNORA70*, *LDHB*, *ITK*, *FYB*, *MIAT*, and *TMEM173* are differentially regulated with large fold changes and expression at relatively high-volume values compared to the findings in conventional T cells (Tables 1 and 2). Surprisingly, most of those genes have not been previously recognized as being specifically regulated in MAIT cells, and their roles are unknown, although several genes, such as *KLRC4* and *KLRB1*, which encode a protein, CD161, have been previously reported^25^. This study strongly highlights our poor knowledge of MAIT cell biology and the importance of identifying the roles of genes in MAIT cells.

MAIT and TCRα7.2^+^ CD161^−^ T cells share an upregulated *ADM* gene as well as the downregulated genes *TNFRSF4*, *FBLN7*, *KIR2DS4*, *WNT7A*, and *ANKRD55* (Supplementary Fig. S1). These findings could provide clues concerning the relationship between MAIT and TCRα7.2^+^ CD161^−^ T cells.

In conclusion, the present findings clarify the differences between MAIT cells, TCRα7.2^+^ CD161^−^ T cells and CD161^−^ conventional T cells and the differences and similarities in the expression of MAIT cells and TCRα7.2^+^ CD161^−^ T cells at the whole genome level. The newly discovered genes that are differentially regulated compared to that in CD161^−^ conventional T cells exemplify our ignorance of MAIT cells and highlight the need for further research.

## Methods

### Cell sorting

Whole blood from ten healthy individuals was obtained from the Red Cross Blood Center. The study protocol was approved by the Institutional Review Board of the Red Cross. For the cell sorting of MAIT cells, CD161^−^ conventional T cells, TCR_α_7.2+ CD161− T cells and total T cells were isolated from the whole blood to >95% purity. This was achieved through negative selection using a RosetteSep Human T Cell Enrichment Cocktail (StemCell Technologies, Vancouver, British Columbia, Canada) and incubating with anti-CD3-APC-Cy7, anti-TCRα7.2-FITC, and anti-CCR161-PE-Cy7 in HBSS supplemented with 4% fetal bovine serum for 15 min. The cells were washed, resuspended in Hank’s Balanced Sal Solution, and sorted into a MAIT cell fraction. Cell subsets were isolated to nearly 100% purity using the FACSARIA III cytometer (BD Biosciences, San Jose, CA, USA.) Cell subsets from three different donors were used for RNA sequencing.

### Sample preparation for Illumina HiSeq sequencing

Total RNA was isolated from cell subsets prepared as described above. An RNA sequencing library was generated using a TruSeq RNA sample preparation Kit v2 according to the user’s instruction manual (Illumina, San Diego, CA, USA). Briefly, mRNA was separated from total RNA using oligo(dT) beads and chemically fragmented. After the double-strand cDNA synthesis of fragmented mRNA, end-repair, adenylation of the 3′ end, and sequencing adapter ligation were performed. This was followed by DNA purification with magnetic beads and PCR amplification. Finally, the amplified library was purified, quantified, and then applied for template preparation. The HiSeq2000 platform was utilized to generate 101-bp paired-end sequencing reads (Illumina).

### Genome mapping and identification of paired-end sequences

All 101-bp paired-end sequence reads were mapped to the full genome sequences for *Homo sapiens* (UCSC hg19) using TopHat version 2.0.13^26^. These mapped reads were merged for each condition (conventional T cell, TCR_α_7.2+ CD161− T cells, and MAIT cell), and transfrags were assembled using Cufflink version 2.2.1^26^. These merged transfrags were quantified for each condition using the Cuffdiff program. Additionally, genes with no mapped reads for each condition were excluded from the analysis. Finally, we identified DEGs that were selected by a fold change cut-off of >2 and an independent T-test raw p-value of <0.05. Finally, RNA-seq analysis revealed 275 DEGs between MAIT cells and conventional T cells and only 20 DEGs between TCRα7.2^+^ CD161^−^ T cells and CD161^−^ conventional T cells. In Fig. 1c, the volume represents the level of gene expression. The volume is calculated by geometric means of mapped reads between two conditions.

### Gene set enrichment test

To characterize the gene set containing DEGs, representative gene sets were analyzed in Ingenuity Pathway Analysis (IPA)^27^. The terminologies of the gene set are listed in the IPA program. Significant tests for the mapping gene sets were performed by Fisher’s exact test (filtering options: p-value ≤ 0.05). In Fig. 3, the significant value of each gene set was represented as −log(p-value). If the p-value is 0.05, −log(p-value) is converted to 1.3.

### Transcription factor analysis

Upstream regulator analysis was conducted to identify the transcription factor of the genes in the dataset that explain the observed differential expression changes in the MAIT cells. Using upstream regulator analysis in IPA, we could determine statistically significant transcription factors of up- and downregulation in MAIT cells. Usually, the upstream factors contain various regulators, such as transcription factors, enzymes, chemicals, and microRNAs. In the analysis, the upstream factor is restricted only to the transcription factor because the transcription factor is the key regulator as an intrinsic factor in the biological pathway. The transcription factors were selected by the cut-off value of p-value ≤ 0.01, which is unlikely to occur in a random model (Supplemental Fig. S1).

### Construction of the biological pathways related to MAIT cells

To construct biological pathways composed of DEGs, we used gene set enrichment tests and interactions derived from the Ingenuity Knowledge Base. Among the several gene sets that were selected as statistically significant pathways in the up- and downregulation of MAIT cells, we focused on immune-related gene sets, such as Th1, Th2, NK, and the macrophage pathway. The DEGs were converted to proteins by matching them to official symbols at Entrez (http://www.ncbi.nlm.nih.gov/Entrez/). To visualize the PPI network, we used the Cytoscape program (http://www.cytoscape.org/). The color of the protein represents the level of fold change by expression in each condition. In Figs 4–6, the box indicates the gene involved in the biological pathway. The red box represents upregulated genes, and the green box shows downregulated genes. The purple box indicates already known important genes in each biological pathway. The information was obtained from the IPA database. A blank (white) box indicates that there is no expression data from the RNA-seq experiments. Solid lines provide evidence of the directed interaction between two genes. However, the dotted line indicates the in-directed interaction.

## Supplementary information

Supplementary figure
