# Supplementary material for: Differences in the molecular signatures of mucosal-associated invariant T cells and conventional T cells
Source: Sci Rep. 2019 May 8;9:7094. doi: 10.1038/s41598-019-43578-9 (PMC6506535; doi:10.1038/s41598-019-43578-9)
Supplement: Supplementary file 1 — Supplementary figure [file 41598_2019_43578_MOESM1_ESM.pdf]

Supplementary Information

Difference in the molecular signatures of mucosal-associated invariant T cells and conventional T cells

Daeui Park, Hong Gi Kim, Miok Kim, Tamina Park, Hyung-Ho Ha, Dae Ho Lee, Kang-Seo Park, Seong Jun Park, Hwan Jung Lim, and Chang Hoon Lee

Supplementary figure 1: Differential gene expression profiles of MAIT cells and TCRVα7.2⁺ CD161⁻ T cells and TCRVα7.2⁻ conventional T cells

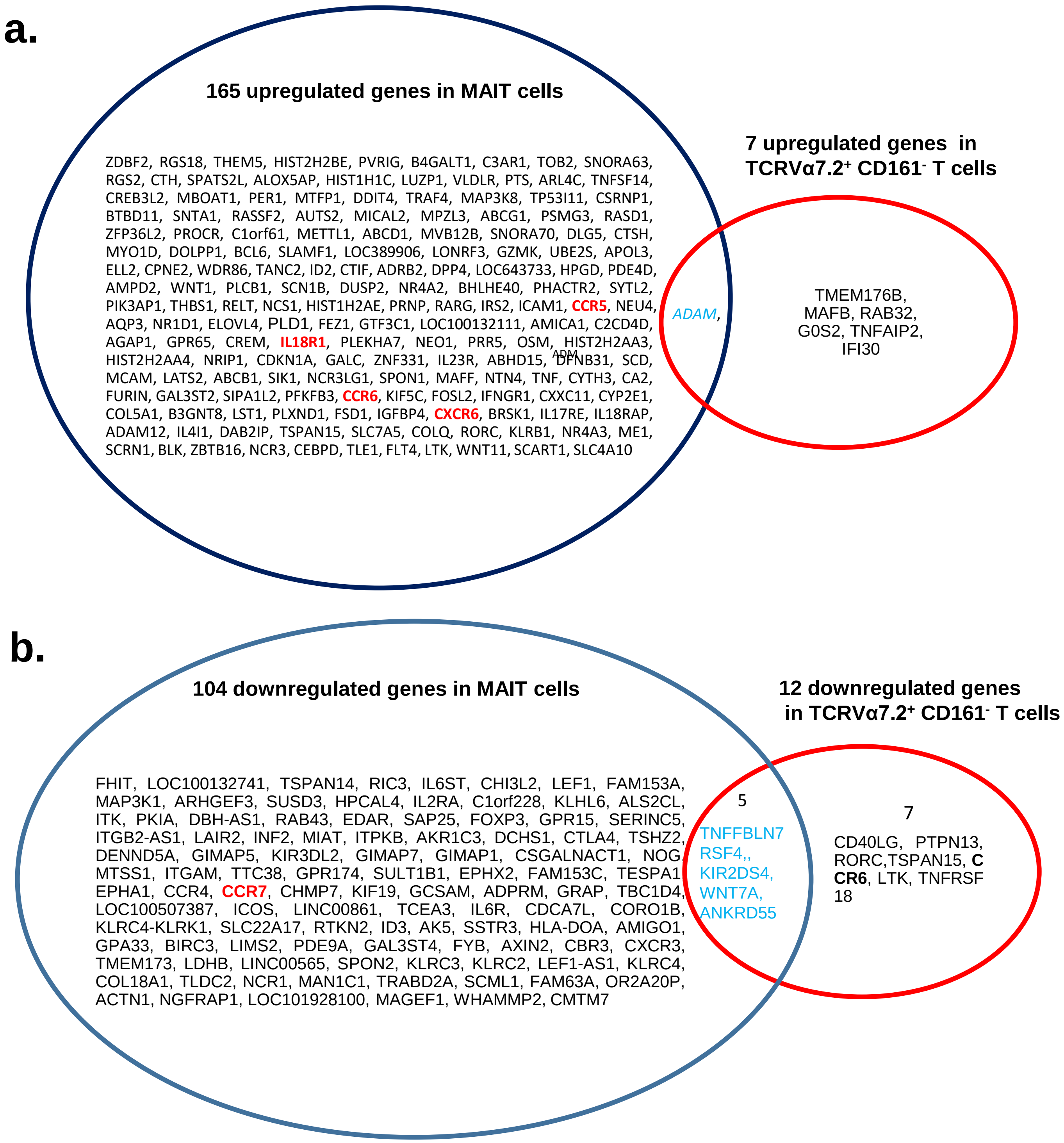

(a) Venn diagram of 165 upregulated DEGs in MAIT cells and 6 upregulated DEGs in TCRVα7.2⁺ CD161⁻ T cells compared to conventional T cells.

(b) Venn diagram of 104 down regulated DEGs in MAIT cells and 7 down regulated DEGs in TCRVα7.2⁺ CD161⁻ T cells compared to conventional T cells.
